# Supplementary material for: Bionomic responses of Spodoptera frugiperda (J. E. Smith) to lethal and sublethal concentrations of selected insecticides
Source: PLoS One. 2023 Nov 15;18(11):e0290390. doi: 10.1371/journal.pone.0290390 (PMC10650980; doi:10.1371/journal.pone.0290390)
Supplement: S9 Table — CT: Control (no insecticide); AZ: NeemGold 0.3 SC® (azadirachtin); BT: Agoo 55WP® (Btk + Monosultap); PR: Bypel 1 WP® (PrGV + Btk); EB: Strike 1.9 EC™ (emamectin benzoate). (DOCX) [file pone.0290390.s009.docx]

**Bionomic responses of *Spodoptera frugiperda* (J. E. Smith) to lethal and sublethal concentrations of selected insecticides**

**Insecticides bio-efficacy on *Spodoptera frugiperda***

**Kokou Rodrigue Fiaboe ^1*^, Ken Okwae Fening^1, 2^, Winfred Seth Kofi Gbewonyo^1, 3^, Sharanabasappa Deshmukh^4^**

**^1^**African Regional Postgraduate Programme in Insect Science (ARPPIS), School of Agriculture, College of Basic and Applied Sciences, University of Ghana, PMB LG 59, Legon, Accra, Ghana.

**^2^**Soil and Irrigation Research Centre, (SIREC), School of Agriculture, College of Basic and Applied Sciences, University of Ghana, P. O. Box LG. 68, Legon, Accra, Ghana.

**^3^**Department of Biochemistry, Cell and Molecular Biology, School of Biological Science, College of Basic and Applied Science, University of Ghana, P. O. Box LG. 68, Legon, Accra, Ghana.

**^4^**Department of Entomology, College of Agriculture, University of Agricultural and Horticultural Sciences (UAHS), Shivamogga-577201, Karnataka, India.

**^*^Corresponding author (**[rfiaboe@yahoo.com](mailto:rfiaboe@yahoo.com)).

**S9 Table** Number of alive Spodoptera frugiperda larvae per ten maize plants per plot during the minor rainy season on-station trial.

CT: Control (no insecticide); AZ: NeemGold 0.3 SC® (azadirachtin); BT: Agoo 55WP® (*Btk* + Monosultap); PR: Bypel 1 WP® (*Pr*GV + *Btk*); EB: Strike 1.9 EC™ (emamectin benzoate).
