# Supplementary material for: Bionomic responses of Spodoptera frugiperda (J. E. Smith) to lethal and sublethal concentrations of selected insecticides
Source: PLoS One. 2023 Nov 15;18(11):e0290390. doi: 10.1371/journal.pone.0290390 (PMC10650980; doi:10.1371/journal.pone.0290390)
Supplement: S10 Table — CT: Control (no insecticide); AZ: NeemGold 0.3 SC® (azadirachtin); BT: Agoo 55WP® (Btk + Monosultap); PR: Bypel 1 WP® (PrGV + Btk); EB: Strike 1.9 EC™ (emamectin benzoate). (DOCX) [file pone.0290390.s010.docx]

**S10 Table** Number of alive *Spodoptera frugiperda* larvae per ten maize plant per plot during the dry season on-station trial.

 CT: Control (no insecticide); AZ: NeemGold 0.3 SC® (azadirachtin); BT: Agoo 55WP® (*Btk* + Monosultap); PR: Bypel 1 WP® (*Pr*GV + *Btk*); EB: Strike 1.9 EC™ (emamectin benzoate).
