# Supplementary material for: Bionomic responses of Spodoptera frugiperda (J. E. Smith) to lethal and sublethal concentrations of selected insecticides
Source: PLoS One. 2023 Nov 15;18(11):e0290390. doi: 10.1371/journal.pone.0290390 (PMC10650980; doi:10.1371/journal.pone.0290390)
Supplement: Attachment — Submitted filename: Response to Reviewers.docx [file pone.0290390.s012.docx]

# Response to Reviewers

# Reviewer #1

**Comment 1:** Brief information of the pesticides used in this study should be mentioned in the introduction.
**Response 1:** The introduction is modified with information on the pesticides used in the study according to the reviewer comment.

**Comment 2:** Line 55: Please briefly introduce S. frugiperda. For example, polyphagous, strong preference to maize, resistance to pesticides, etc.

**Responses 2:** Lines 56 – 63: Revision made according to the reviewer comment

**Comment 3:** Line 115: Why castor bean leaves instead of maize leaves were used in the rearing? Different diets of S. frugiperda might alter the physiological status of this pest and therefore change their resistance to pesticides.

**Response 3:** In our research, the decision to rear *S. frugiperda* larvae on castor bean leaves instead of maize leaves was based on practical considerations. Castor bean leaves have been widely used as a suitable diet for rearing fall armyworm in laboratory settings due to their availability, nutritional value, and support for larval development. This choice allowed us to maintain a controlled and consistent rearing environment, ensuring uniformity in the developmental stage and physiological status of the tested insects. However, we acknowledge that there is a potential discrepancy between the rearing diet and the choice of maize leaves during the leaf-dip bioassays. It is possible that the use of different diets could influence the physiological responses and resistance mechanisms of *S. frugiperda* to the tested pesticides. However, the use of maize leaves consistently during the bioassays isolate any potential influences of the diet on the bioactivity of the insecticides, providing a clearer understanding of the insect's susceptibility to the tested pesticides.

**Comment 4:** Line 225: Homogeneity of variance test and normality test should be mentioned before ANOVA analysis.

**Response 4:** Lines 229 – 231: Statement on homogeneity of variance and normality tests is included according to the reviewer’s comment.

**Comment 5:** Line 233: full name of HAT (hours after treatment) should be mentioned in the first time.

**Response 5**: Line 243: “(hours after treatment)” mentioned after HAT

**Comment 6:** Table 2: the annotation of “N”

**Response 6:** Line 264: “N” defined.

**Comment 7:** Table 3: “Fecundity” means number of eggs produced by individual female insect?

**Response 7**: Line 178: For clarity, the term “Fecundity” is defined when mentioned for the first time in the text.

**Comment 8:** Table 7 and 8: Compared with number of alive larvae, it might be more reasonable to compare the survival rate of S. frugiperda larvae after pesticide spray?

**Response 7**: Tables 7 and 8 are replaced with Figures 3 and 4, presenting the population reduction.

**Reviewer #2:**

**#** The language of this paper needs to be improved; it is better to reduce the qualitative statement in the result description through this study.

**Response:** The language was revised accordingly.

**#** The unit ppm could not indicate the connection of solution intuitively, please use mg/L or g/L.

**Response:** ppm is replaced with mg/L according to review suggestion.

**#** Study location is not necessary or this part could be introduced in methods in field.

**Response:** “Study location” is relocated to on-station experiment section

**#** Line 170, Ten (10) or Line Two (2) revised to Ten or Two.

**Response:** Correction made accordingly.

\# Line 116, the relative humidity misses the percent sign % after the number.

**Response:** Correction made accordingly.

________________________________________
